# Effects of screw-pressing temperature on the functional properties and structural characteristics of apricot (*Prunus armeniaca* L.) kernel protein isolates

**DOI:** 10.3389/fnut.2025.1619072

**Published:** 2025-06-30

**Authors:** Li Zhang, Hongyu Wu, Mengshi Wang, Xianjin Zhou, Bayinkexike, Ruiguo Cui, Lijun Song, Fengjuan Liu

**Affiliations:** ^1^College of Food Science and Technology, Hebei Normal University of Science and Technology, Qinhuangdao, China; ^2^Hebei Key Laboratory of Natural Products Activity Components and Function, Hebei Normal University of Science and Technology, Qinhuangdao, China; ^3^Bazhoujiamu Agroscience Co., Ltd., Korla, Xinjiang, China; ^4^Development Exchange Center of Bayingol Mongolian Autonomous Prefecture, Korla, Xinjiang, China; ^5^Institute of Quality Standards and Testing Technology for Agro-Products, Xinjiang Academy of Agricultural Sciences, Urumqi, China

**Keywords:** apricot kernel protein isolate, functional properties, structural characteristics, screw-pressing temperatures, multivariate statistical analysis

## Abstract

This study aimed to investigate the effect of screw-pressing temperature on the quality of apricot kernel protein isolates (API). The API values at different screw-pressing temperatures (40–200°C) were obtained, and the functional and structural properties of different API samples were comparatively studied. The results revealed that the total polyphenol content (TPC), total flavonoid content (TFC), and antioxidant activities (DPPH and FRAP assays) increased significantly with increasing temperature. High-temperature pressing also increased the surface hydrophobicity and emulsification of API. SDS-PAGE confirmed the preservation of the primary structure of API, with molecular weights ranging from 13 to 20 kDa and 36–56 kDa. Circular dichroism (CD) spectroscopy analysis revealed that the *α*-helix content increased (by 4–8%) and the *β*-sheet content decreased (by 2–5%) when the samples were pressed at high temperatures. The decrease in fluorescence intensity and the fluorescence spectral shift indicated changes in the tertiary structure. Multivariate statistical analysis revealed that the antioxidant activities were positively correlated to protein carbonyls, free sulfhydryl groups, surface hydrophobicity, TPC, and TFC. Mechanistically, thermally-induced protein conformational changes and surface hydrophobicity modulation drove the observed enhancements in functional properties. These findings will collectively serve as a theoretical basis for the efficient preparation and application of API.

## 1 Introduction

Apricot kernels are homologs of medicinal and food materials that are rich in oil, protein, and various functional active substances, such as tocopherols, polyphenols, and polyunsaturated fatty acids (1, 2). After oil extraction, the protein content in the residue can reach as high as 68%, rendering apricot kernels good candidates for protein supplementation in food and nutraceutical applications (3). Apricot kernel protein isolate (API) exhibits important flavor characteristics and functional properties, such as foaming ability and emulsifying ability (4). Additionally, API possesses various biological properties, such as immunomodulatory, antitumor, and antioxidant properties. Therefore, API can be used in functional foods, dietary supplements, and pharmaceutical products (5).

The functional and biological properties of proteins are affected by multiple factors, such as protein structure, surface properties, amino acid composition, and extraction and processing conditions, including pH, ionic strength, temperature, etc. (6). Currently, apricot kernel protein is produced mainly from the cake and meal through mechanical oil pressing (3). However, during the spiral pressing process, thermomechanical action (high temperature, high pressure, and high shear) may lead to structural changes in the protein, causing significant changes in the protein’s functional and biological properties (7). Temperature, in addition to structure and functional characteristics, is an important factor affecting proteins (8). In rice protein, for example, notable decreases in the contents of sulfhydryl groups, disulfide bonds, and hydrogen bonds were observed as the structural changes upon screw pressing (9). High-temperature extrusion was also reported to cause the formation of structured protein aggregates in rice protein (9). Similar results were reported for buckwheat globulin (10) and rice bran protein isolate (11). Moreover, moderate heat treatment (60°C and 80°C) caused the partial unfolding and aggregation of lotus seed protein and increased the average diameter and surface hydrophobicity (12). In terms of functional property changes, moderate heat treatment (60–80°C) significantly enhanced the solubility, water/oil holding capacity, emulsification, and foaming characteristics in quinoa albumin and promoted sulfhydryl-disulfide interchange and exposure of hydrophobic groups (7). Moreover, radio frequency-based heating treatment (above 100°C) improved both absorption and emulsifying properties of rice bran protein isolate (11). Heating at 100°C for 30 min caused band splitting and maximum unfolding while decreasing the available lysine residues in the album protein isolates, simultaneously improving the thermal stability and *in vitro* digestibility (which increased to 87.55%) (13). The existing research has focused mostly on protein extraction or quality at a single temperature, and studies on the changes in the functional properties and structural characteristics of API under different screw-pressing temperatures are lacking.

In this study, the effects of different screw-pressing temperatures on API quality were investigated. The physicochemical properties (color, antioxidant activities, total polyphenol, and total flavonoid contents), functional properties (absorption, emulsification, and surface properties), and structural characteristics (secondary and tertiary structures) of API at different screw-pressing temperatures (40–200°C) were evaluated and compared. Multivariate statistical analysis was conducted to determine the relationships between the tested indicators of different API samples. The findings will serve as a theoretical basis for the preparation and application of high-quality API.

## 2 Materials and methods

### 2.1 Materials and reagents

Youyi sweet apricot kernels (*P. armeniaca* L.) were procured from Zhangjiakou City, Hebei Province, China, on August 20, 2024.

Folin was purchased from Yuanye Biotechnology Co. Ltd. (Shanghai, China). The bicinchoninic acid protein concentration determination kit (Enhanced) was obtained from Beyotime (Shanghai, China). The main reagents used in this study were 2,4-dinitrobenzoic acid, trichloroacetic acid, guanidine hydrochloride, urea, aluminum chloride, potassium hydroxide, hydrochloric acid, anhydrous ethanol, ethyl acetate, n-hexane, sodium hydroxide, methanol, sodium carbonate, sodium dihydrogen phosphate, disodium hydrogen phosphate, gallic acid, rutin, 8-ani-lino-1-naphthalenesulfonic acid (ANS), Tris aminomethane, 2,2-diphenyl-1-picrylhydrazyl (DPPH), total antioxidant capacity test kits, SDS-PAGE gel preparation kits, and Coomassie brilliant blue staining reagents. All these reagents were purchased from Solarbio Technology Co. Ltd. (Beijing, China).

### 2.2 Preparation of the apricot kernel protein isolate

After the outer crusts of the apricot stone were removed, the kernels were air-dried and stored at 4°C until use. The apricot kernel cakes were prepared using a screw press (LBT01, Foshan Liangtai Optoelectronics Technology Co. Ltd., China) and then degreased through pressing at five different screw-pressing temperatures (40°C, 80°C, 120°C, 160°C, and 200°C). The resulting apricot kernel cakes were collected and used for the preparation of API using the alkaline acid precipitation method (8) with slight modifications. First, the apricot kernel cake was ground into a fine powder (50-mesh) followed by defatting using hexane. Then, the degreasing powder (45.0 g) was dissolved in distilled water (450 mL), and its pH was adjusted to 9.0 using NaOH solution (2.0 mol/L). The mixture was subjected to magnetic stirring at room temperature for 1 h and then centrifuged for 15 min at 4°C and 6,000 r/min. The supernatant was collected, and its pH was adjusted to 4.5 using an HCl solution. The mixture was allowed to stand still to separate the protein. The turbid protein was then collected and centrifuged for 15 min at 4°C and 6,000 r/min. The protein precipitate was collected and washed three times with deionized water, after which the pH was adjusted to 7.0 using NaOH solution. Finally, the obtained API samples were freeze-dried and stored at 4°C until used.

### 2.3 Functional property analysis

The water-soluble protein content, nitrogen soluble index (NSI), water-holding capacity (WHC), and oil-holding capacity (OHC) were determined as described in previous reports (14). The emulsifying activity index (EAI) and emulsion stability index (ESI) were calculated using previously reported methods (15). The details of the measurement method (Method S1) are provided in the Supplementary materials.

### 2.4 Physicochemical property analysis

#### 2.4.1 Color

The color of each sample was determined using a spectroscopic color measuring device (YS6003, 3nh Co. Ltd., Shenzhen, China) and represented using the CIE Lab chromaticity indicators, including L (lightness), a (redness), and b (yellowness).

#### 2.4.2 Total polyphenol content (TPC) and total flavonoid content (TPC)

The TPC and TFC values for the samples were determined using previously published protocols (16) and expressed in gallic acid (mg GAE /g·dw) equivalents and rutin equivalents (mg RE/g·dw) units, respectively.

#### 2.4.3 Antioxidant activity

The total antioxidant capacity test kit (Solarbio Technology Co. Ltd., Beijing, China) was employed to determine the ferric ion-reducing antioxidant power (FRAP) according to the manufacturer’s instructions.

DPPH activity was measured using a previously reported method (16). In brief, the API solution (0.20 mL) was mixed with the DPPH solution (0.95 mL) and pure methanol (2.85 mL) in a test tube and allowed to react in the dark for 30 min. Afterward, the absorbance of the reaction mixture was measured at 517 nm. The results were expressed as mg GAEAC (g·dw)^−1^, and the values were calculated using the following formula:


$$\text{DPPH assay}\;(\mathrm{mg}\;\text{GAEAC}{\left(g\cdot \mathrm{dw}\right)}^{-1})=\frac{\Delta {\mathrm{Abs}}_{\text{sample}}}{\Delta {\mathrm{Abs}}_{\text{standard}}}\times C\times \frac{V}{W}$$

where, C denotes the concentration of gallic acid (mg/mL), V denotes the volume of sample (mL), and W denotes the weight of the powdered sample (g).

### 2.5 Structural characterization

#### 2.5.1 Basic structural composition

Protein carbonyls were determined using a previous method (16). The free sulfhydryl content and surface hydrophobicity were analyzed according to previously reported methods (17). The amino acid composition was determined using a previously published method (18). The details of these determination methods are provided in the Supplementary materials (Method S2).

#### 2.5.2 SDS-PAGE

An SDS-PAGE gel preparation kit was used according to the manufacturer’s instructions. The API powder was dissolved in Tris HCl buffer solution (10 mmol/L) to prepare the protein solution (5 mg/mL). Next, the protein solution and buffer solution were heated in a boiling water bath for 5 min and then centrifuged at 14000 × g for 5 min to remove the insoluble components. The concentrations of the separation gel and concentration gel were 12 and 5%, respectively. The treated protein mixture (5 μL) was subsequently applied to the lanes in the gels (prefabricated acrylamide, 4–20%), and electrophoresis was performed using a vertical electrophoresis apparatus (Power BV, Beijing Kaiyuan Xinrui Instrument Co. Ltd., China) at an initial voltage of 80 V, which was later adjusted to 120 V when the strip was moved to the separation gel. After electrophoresis, the protein bands were stained with Thomas brilliant blue R-250 followed by multiple rounds of decolorization. After decolorization, a gel imaging system was used to scan the strips (17).

#### 2.5.3 Fluorescence spectrogram

In accordance with previously published methods (19), the fluorescence spectra of API (2.0 mg/mL) were obtained using a fluorescence spectrophotometer (F-4500, Hitachi, Japan) operated at a voltage of 700 mV, an excitation wavelength of 290 nm, and an emission wavelength of 300–400 nm. The slit width was 5 nm, the scanning speed was 200 nm/min, and the increment was 10 nm.

#### 2.5.4 CD spectroscopy

In accordance with the previously published methods (16), CD spectroscopy of the API solution (0.2 mg/mL in PBS, pH = 8) was performed using a spectropolarimeter (Chrascan 100, Applied Photophysics Ltd., UK). The scan speed was 50 nm/min, the spectral resolution was 0.1 nm, the response time was 0.1 s, and the bandwidth was 1 nm.

#### 2.5.5 Particle size and zeta potential

The particle size distribution and the zeta potential of each API sample (0.01 mol/L) were determined using a Malvern Zetasizer Nano instrument (ZS90, Shanghai Sibaiji Instrument System Co. Ltd., China). The refractive index was 1.46 (17).

#### 2.5.6 Scanning electron microscopy (SEM)

The microscopic morphology of the API samples was recorded using SEM (SU8010, Hitachi Corporation, Japan) (14). After gold coating, samples were subjected to SEM analysis at an accelerating voltage of 20 kV and a magnification of 500–3,000, and images were recorded.

### 2.6 Statistical analysis

Each experiment was performed three times, and the results were expressed as means ±standard deviations. Origin 2021 software (Origin Lab Corp., Northampton, UK) was used for multivariate statistical analysis and statistical chart generation. SPSS software (SPSS Statistics 27, IBM, US) was used to conduct one-way and type I analysis of variance (ANOVA) tests. Duncan’s multiple-range tests were conducted to assess the parameter differences between the treatment groups.

## 3 Results and discussion

### 3.1 Functional properties

**Figure 1:**
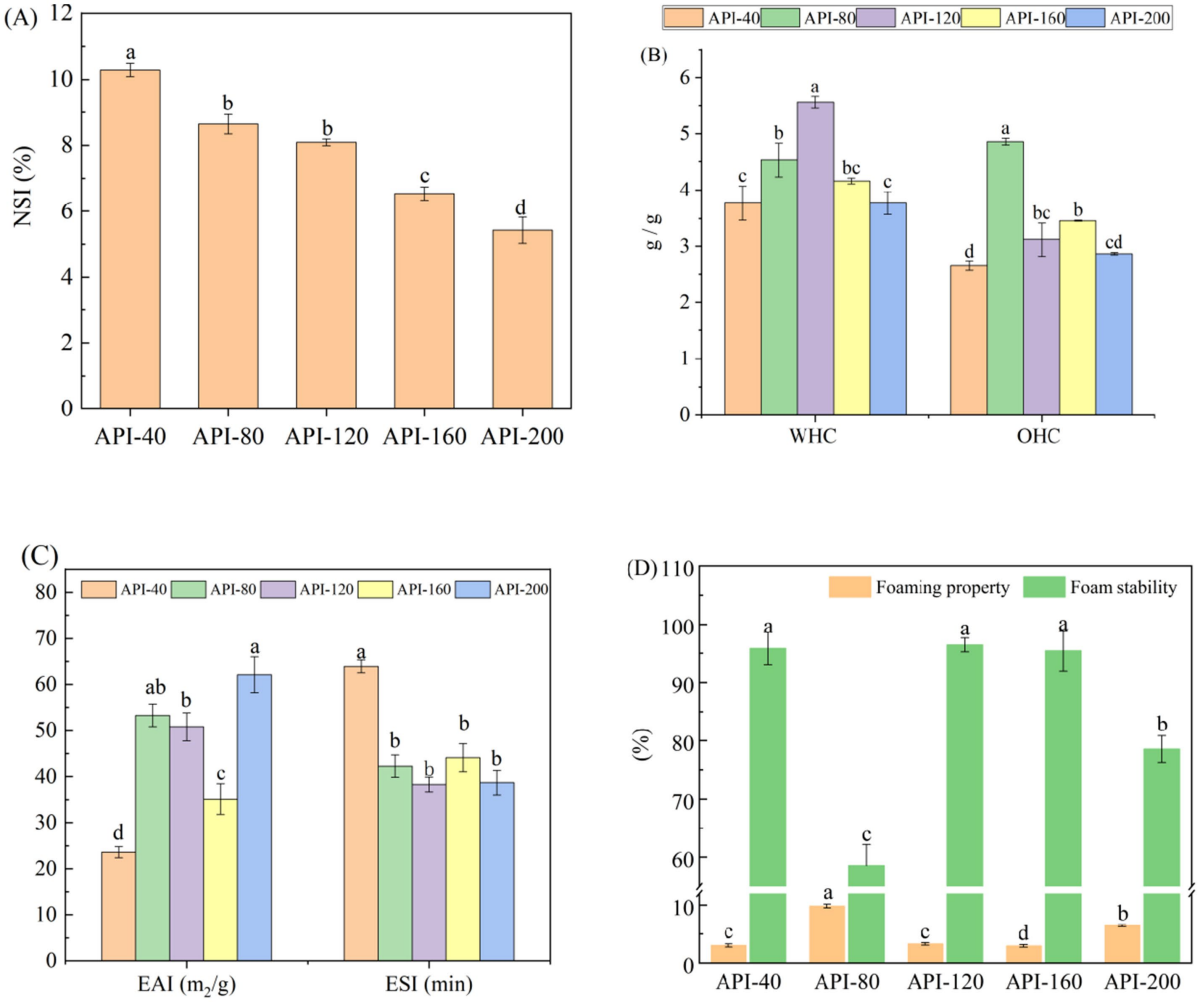
The functional characteristics of API at different screw-pressing temperatures. **(A)** NSI; **(B)** WHC and OHC; **(C)** EAI and ESI; **(D)** Foaming properties and stability. NSI, Nitrogen soluble index; WHC, Water-holding capacity; OHC, Oil-holding capacity; EAI, Emulsifying activity index; ESI, Emulsion stability index.

Figure 1 shows the changes in the functional characteristics of different API samples. The screw-pressing temperature significantly affected the functional characteristics (*p* < 0.05). As shown in Figure 1A, the NSI values of API clearly decreased with increasing temperature (*p* < 0.05). This could be because the protein was modified under the combined effects of temperature, shear force, and pressure during the extrusion process (9). Moreover, heat causes denaturation and damage to the lysine, arginine, and cysteine residues, which lowers protein solubility (20).

As shown in Figure 1B, appropriate hot pressing improved the WHC and OHC of the API samples. The WHC and OHC of the control sample (API-40) were 3.77 and 2.65 g/g, respectively. However, API-120 presented the highest WHC (5.56 g/g), whereas API-80 presented the highest OHC (4.86 g/g). Similar results have been reported for defatted moringa seed flour (21) and wheat flour (22). According to a previous study, high temperatures and pressures can stretch peptide chains and expose the hydrophilic groups, thereby improving hydration ability (14). As shown in Figure 1C, the EAI (ranging from 23.61 to 62.11 m^2^/g) and ESI (ranging from 38.29 min to 63.95 min) of different API samples exhibited significant differences (*p* < 0.05). The reason for this could be that high temperature, pressure, and shearing change the protein structure, including changes in protein unfolding, aggregation, and rearrangement. These structural changes are capable of affecting the distribution sites of different groups (hydrophilic and hydrophobic groups). Therefore, the surface hydrophobicity (as shown in Table 1) and emulsification efficiency of API improved (9).

**Table 1:** Color, TPC, TFC, and antioxidant properties of API at different screw-pressing temperatures.

Sample	API-40	API-80	API-120	API-160	API-200
Sample picture	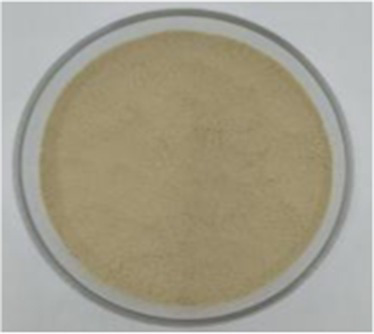	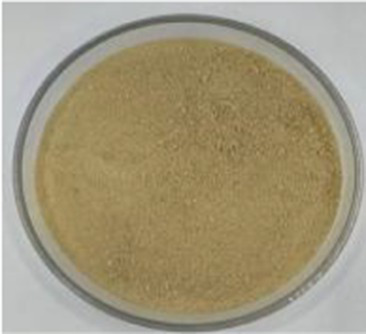	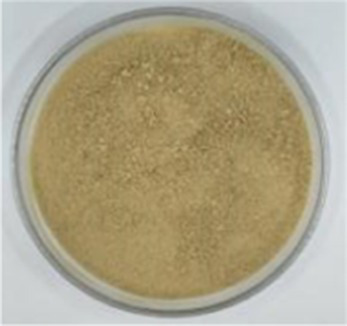	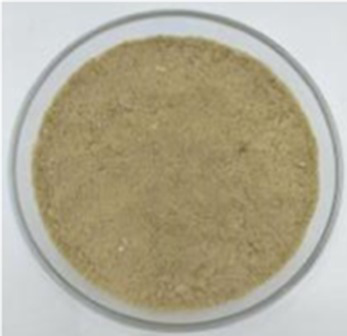	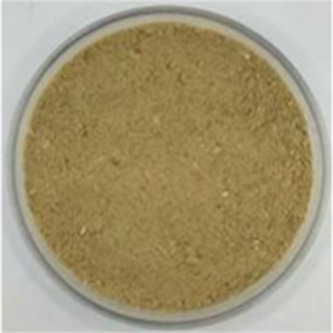
L	56.00 ± 4.07^a^	43.44 ± 2.67^b^	5.14 ± 2.61^d^	11.55 ± 2.8^c^	7.81 ± 0.09^cd^
a	3.87 ± 0.07^c^	5.03 ± 0.11^c^	24.22 ± 0.67^a^	14.55 ± 2.88^b^	22.26 ± 2.59^a^
b	15.24 ± 0.37^b^	20.23 ± 0.58^a^	10.91 ± 2.95^c^	16.27 ± 0.65^ab^	16.69 ± 3.47^ab^
TPC (mg GAE/g dw)	1.19 ± 0.15^d^	1.29 ± 0.21^c^	1.33 ± 0.16^bc^	1.39 ± 0.11^ab^	1.42 ± 0.13^a^
TFC (mg RE/g dw)	19.57 ± 0.78^c^	22.67 ± 0.71^b^	27.97 ± 0.53^b^	27.81 ± 0.34^a^	35.60 ± 0.51^a^
DPPH (mg GAEAC/g dw)	5.22 ± 0.68^e^	5.81 ± 0.35^d^	10.43 ± 0.42^c^	10.71 ± 0.73^b^	14.36 ± 0.86^a^
FRAP (μmol Trolox/g)	139.84 ± 9.98^c^	203.58 ± 10.05^b^	201.69 ± 11.2^b^	245.44 ± 10.55^b^	342.22 ± 11.27^a^

TPC, total polyphenol content; TFC, total flavonoid content; DPPH value, 2,2-diphenyl-1-picrylhydrazyl radical scavenging activity; FRAP value, ferric ion-reducing antioxidant power. Different letters indicate statistical difference among groups (*p* < 0.05).

As shown in Figure 1D, the foaming properties and foam stability significantly differed among different API samples. API-80 presented the greatest foaming properties and lowest foam stability. Previous studies have reported that during high-temperature pressing, certain proteins undergo denaturation, exposing the hydrophobic groups and decreasing the ability of the proteins to bind to water molecules (9). This may lead to a reduced capacity to form multiple bubble spaces during homogenization, decreasing the foaming ability of API and increasing its foam stability (9).

### 3.2 Phytochemical analysis

#### 3.2.1 Color

Table 1 presents the results of the calorimetric analysis (values of L, a, and b) of different API samples. As the temperature increased, the L values (lightness) of different API samples decreased significantly. The ‘L’ values of the control sample (API-40) and API-200 were 56.00 and 7.81, respectively, while the ‘a’ values (redness) ranged from 3.78–24.22, and the ‘b’ values (yellowness) were distributed between 10.91 and 20.23. These results were similar to those reported in previous studies for walnut protein (23). The color changes noted in this study may be due to the Maillard reaction between amino acids and reducing sugars, as well as due to the oxidation of phenolic compounds caused by high temperatures (23).

#### 3.2.2 TPC and TFC

Table 1 shows the TPC and TFC for different API samples. Both TPC and TFC significantly increased with increasing screw-pressing temperature. While TPC increased from 1.19 to 1.42 mg GAE/g· dw, TFC increased from 19.57 to 35.60 mg RE/g· dw. A comparable result was reported in the study on partially defatted moringa seed flour, in which the sample obtained at 200°C presented the highest TPC and TFC (21). As reported previously, the high temperatures used in screw pressing could have broken the bonds of the bound phenolic fraction, which increased the quantity of free polyphenols (21). Another possible explanation could be the partial degradation of lignin, leading to the release of phenolic acid derivatives (24). For example, heat may have caused the disintegration of gallate derivatives and their conversion into gallic acid (24).

#### 3.2.3 Antioxidant activity

As displayed in Table 1, hot pressing also increased the antioxidant activity of different API samples. When the temperature exceeded 120°C, there was a significant increase in the DPPH value. The DPPH value of the control sample was 5.22 mg GAEAC (g·dw)^−1^, and the DPPH values of API-120, API-160, and API-200 were as high as 10.43, 10.71, and 14.36 mg GAEAC (g·dw)^−1^, respectively. In particular, API appeared to have greater antioxidant capacity than the other degreasing seeds, such as partly defatted chia flour (2.58 mg GAEAC (g·dw)^−1^) (25) and degreased sesame seeds (0.78 mg GAEAC (g·dw)^−1^) (26). Similarly, compared to API-40 (139.84 mg GAEAC (g·dw)^−1^), hot pressing also increased the FRAP value. When the samples were pressed at temperatures higher than 80°C, the FRAP values exceeded 203.58 μmol Trolox/g. When the temperature reached 200°C, the FRAP value of API-200 was as high as 342.22 μmol Trolox/g. In a previous study, high-temperature pressing enhanced the antioxidant capacity of legume protein (27). The high content of polyphenols and flavonoids could be a reason for the increased antioxidant activity of API (28). Additionally, high temperatures promote the production of Maillard reaction products, which also exhibit a certain degree of antioxidant activity (28).

### 3.3 Structural characteristics

#### 3.3.1 Basic structural composition

Protein carbonyls are carbonyl-containing compounds generated through the oxidative modification of side chains in certain specific amino acid residues (e.g., lysine, arginine, proline, and threonine) within protein molecules. The content of protein carbonyls is indicative of the degree of protein oxidation (29). Table 2 shows the protein carbonyls, free sulfhydryl groups, and surface hydrophobicities of the different API samples in this study.

**Table 2:** Protein carbonyls, free sulfhydryl groups, and surface hydrophobicities of different API samples.

Sample	API-40	API-80	API-120	API-160	API-200
Protein carbonyls (nmoL/mg)	3.14 ± 0.2^c^	3.33 ± 1.39^bc^	3.86 ± 1.02^bc^	4.67 ± 0.2^ab^	5.47 ± 0.73^a^
Protein sulphydryl (μmoL/g)	8.03 ± 0.29^a^	7.82 ± 0.22^ab^	7.62 ± 0.21^b^	4.63 ± 0.12^d^	5.22 ± 0.12^c^
Surface hydrophobicity (H_0_)	163.39 ± 10.45^e^	211.91 ± 10.91^d^	264.11 ± 9.38^c^	312.61 ± 11.41^b^	380.21 ± 9.68^a^

Different letters indicate statistical difference among groups (*p* < 0.05).

As indicated in Table 2, the protein carbonyl content in the five API sample types increased significantly with increasing screw-pressing temperature (*p* < 0.05). API-200 (5.47 nmol/mg) contained more carbonyl groups than API-40 (3.14 nmol/mg). This phenomenon indicated that high temperatures promote the oxidation reactions of proteins, causing more amino acid residues to be oxidized. A similar result was reported in a study on soy protein (30).

For the free sulfhydryl groups (Table 2), the low-temperature samples presented more free sulfhydryl groups than the high-temperature samples did (8.03 μmol/g (API-40) vs. 4.63 μmol/g (API-160)). Similar results have been reported for rapeseed protein isolates, in which heat treatment resulted in progressive decreases in the free sulfhydryl groups (31). This reduction might be explained by the following mechanisms. First, heat treatment results in different degrees of aggregation or unfolding of proteins, altering the content of free sulfhydryl groups (31). In addition, during heat treatment, sulfhydryl groups may be converted into disulfide bonds through oxidation reactions (32). Third, free sulfhydryl groups may react with the active groups of amino acid residues (such as the *ε*-amino group of lysine) to form covalent bonds (such as thioether bonds) (32).

Surface hydrophobicity is defined as the extent to which a protein’s hydrophobic regions are exposed on its surface, and this parameter is related to the solubility and structure of the protein (17). As shown in Table 1, with increasing screw-pressing temperature, the surface hydrophobicities of the different API samples also increased significantly, ranging from 163.39 (API-40) to 380.21 (API-200). During the high-temperature pressing process, the protein undergoes denaturation, resulting in broken peptide bonds and altered amino acid side chain groups, which causes protein molecules to be cleaved, polymerized, and aggregated. This ultimately reduces the exposure of hydrophilic groups and increases the exposure of hydrophobic groups. This may be the main reason for the increased surface hydrophobicity and decreased solubility of API observed in this study. A previous study revealed that when soy protein was thermally denatured, the proteins aggregated primarily through non-covalent interactions, which also resulted in decreased solubility (33). A similar result was reported for extruded modified rice protein (9).

The contents of 17 amino acids in API were determined, and the results are listed in Table 3. The amino acid composition of API was complete and rich in content and was significantly affected by the screw-pressing temperature (*p* < 0.05).

**Table 3:** Amino acid composition of different API samples (g/100 g).

Sample	API-40	API-80	API-120	API-160	API-200
Essential amino acids					
Lys	2.17 ± 0.04^a^	1.80 ± 0.03^b^	2.13 ± 0.08^a^	1.54 ± 0.02^c^	1.83 ± 0.09^b^
Met	0.18 ± 0.02^c^	0.21 ± 0.03a^b^	0.27 ± 0.03^a^	0.12 ± 0.09^d^	0.13 ± 0.06^d^
The	2.26 ± 0.06^a^	1.85 ± 0.05^d^	2.22 ± 0.05^b^	1.68 ± 0.03^e^	2.07 ± 0.06^c^
Phe	4.53 ± 0.13^b^	4.04 ± 0.03^c^	4.84 ± 0.01^a^	3.70 ± 0.04^d^	4.84 ± 0.02^a^
Val	3.92 ± 0.04^c^	3.41 ± 0.09^d^	4.11 ± 0.03^a^	3.11 ± 0.06^e^	4.02 ± 0.02^b^
Leu	5.83 ± 0.15^b^	5.03 ± 0.08^c^	6.02 ± 0.05^a^	4.56 ± 0.09^d^	5.89 ± 0.04^b^
IIe	3.05 ± 0.03^c^	2.66 ± 0.05^d^	3.20 ± 0.04^a^	2.42 ± 0.09^e^	3.13 ± 0.10a^b^
Total	21.94 ± 4.43^a^	19.00 ± 0.39^b^	22.79 ± 0.29^a^	17.13 ± 0.42^c^	21.91 ± 0.3^a^
Non-essential amino acids					
Gly	3.83 ± 0.06^b^	3.39 ± 0.07^c^	4.06 ± 0.11^a^	3.00 ± 0.10^d^	4.03 ± 0.10^a^
Ala	3.86 ± 0.09^a^	3.36 ± 0.02^b^	3.97 ± 0.03^a^	3.11 ± 0.07^b^	3.98 ± 0.06^a^
Ser	3.15 ± 0.09^a^	2.61 ± 0.06^c^	3.09 ± 0.08^ab^	2.49 ± 0.09^d^	3.21 ± 0.07^a^
Cys	0.21 ± 0.10^d^	0.26 ± 0.08^d^	0.33 ± 0.10^b^	0.29 ± 0.04^c^	0.44 ± 0.01^a^
Asp	8.51 ± 0.16^c^	7.77 ± 0.02^d^	9.23 ± 0.05^b^	7.04 ± 0.04^e^	9.43 ± 0.17^a^
Glu	17.00 ± 0.03^b^	15.9 ± 0.06^c^	18.8 ± 0.02^a^	14.6 ± 0.08^c^	19.9 ± 0.60^a^
Arg	7.66 ± 0.02^c^	6.90 ± 0.04^d^	8.24 ± 0.05^b^	6.42 ± 0.02^e^	8.61 ± 0.16^a^
Tyr	2.50 ± 0.05^a^	2.09 ± 0.02^c^	2.58 ± 0.09^a^	1.84 ± 0.04^d^	2.48 ± 0.09^ab^
Pro	3.47 ± 0.06^b^	3.12 ± 0.04^c^	3.70 ± 0.07^a^	2.83 ± 0.02^d^	3.62 ± 0.08^a^
His	1.81 ± 0.04^b^	1.63 ± 0.09^c^	1.96 ± 0.05^a^	1.48 ± 0.10^d^	1.91 ± 0.04^a^
Total	52.00 ± 0.7^b^	47.03 ± 0.45^c^	55.96 ± 0.65^a^	43.10 ± 0.6^d^	57.61 ± 1.28^a^

Different letters indicate statistical difference among groups (*p* < 0.05).

The API samples contained a relatively reasonable composition of essential amino acids (EAA). The total content of EAA did not change significantly overall, ranging from 17.13 to 22.79 g/100 g, whereas API-160 presented the lowest content (17.13 g/100 g). Notably, EAA accounted for a high proportion of total amino acids (TAA), with a range of 38.08–42.19%. This proportion was significantly greater than that reported for soy protein (about 34%) (34). Among all EAAs, Leu, Phe, and Val were the top three amino acids in all samples. The content of lysine (Lys) significantly decreased under high-temperature pressing (*p* < 0.05). The most susceptible amino acid during extrusion is Lys, with a high retention rate reported in mung bean protein after extrusion (18). In addition, under high-temperature conditions, lysine is prone to the Maillard reaction with reducing sugars, which leads to a decrease in the effective content of lysine (35). However, the content of methionine (met) in API in this study was relatively low, with a value of less than 0.27 g/100 g. This result was similar to that reported for soy protein, which was the first limiting amino acid (less than 1.0 g/100 g) to be reported (34).

The content of non-essential amino acids (NEAA) ranged from 43.10 to 57.61 g/100 g, with glutamate (Glu), aspartate (Asp), and arginine (Arg) as the most abundant amino acids. This finding is consistent with the findings of a previous study (4). Glu is the basic amino acid of nitrogen metabolism in organisms and can also be used as a food additive to improve the taste and quality of food products. Asp is widely used in the food and medical industry owing to its ability to relieve fatigue and promote mineral absorption. Arg offers cardiovascular protection and immune activation functions (36). The contents of hydrophobic amino acids, including Val, Gly, Leu, and Ala, in API-120, were greater than those in the other API samples, which may be due to the intra-and intermolecular interactions that occurred during the extrusion treatment (37).

#### 3.3.2 Particle size distribution and zeta potential

**Figure 2:**
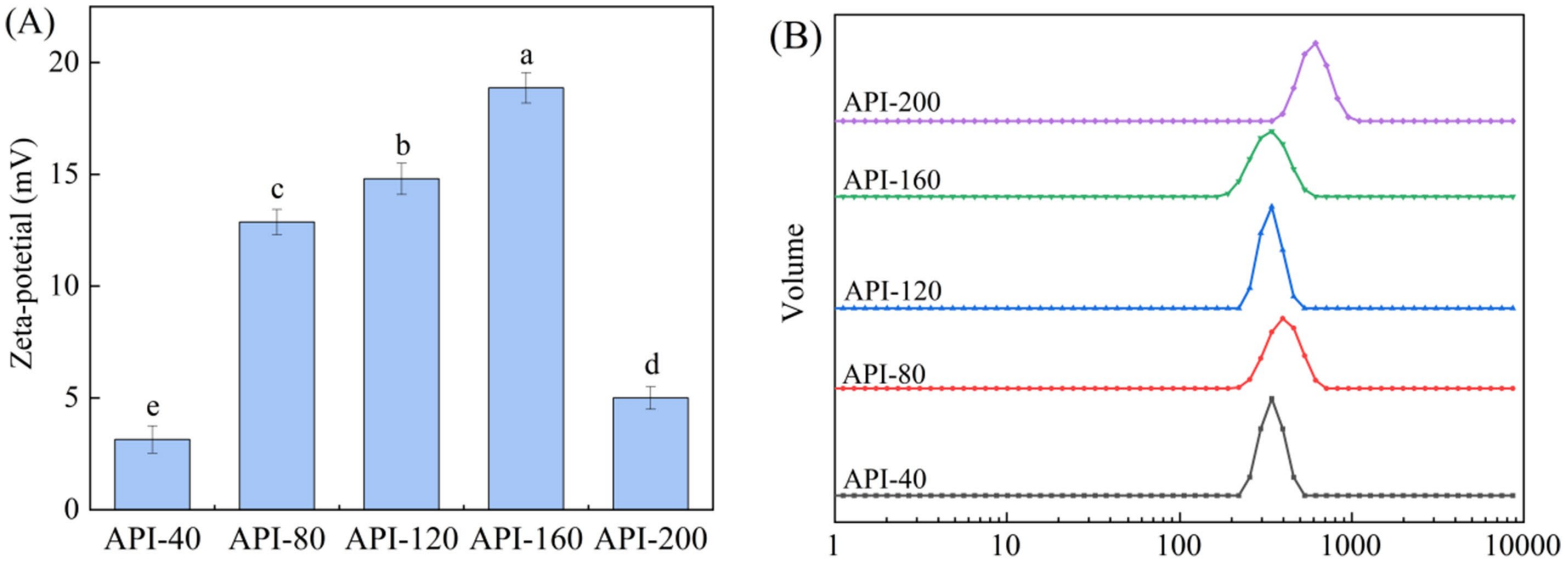
Particle size distribution **(A)** and zeta-potential **(B)** of different API types.

The zeta potential, which is determined directly by the quantity and polarity of the charges carried by amino acids located on the protein surface, is a reliable indicator of the stability of the protein solution dispersion system (17). As illustrated in Figure 2A, the zeta potential values of all five API types were positive, indicating that there were more positively charged amino acids than negatively charged amino acids present on the protein surface (17). The protein solution stabilizes the solution system through intermolecular electrostatic repulsion; the larger the absolute value of the zeta potential, the more homogeneous the charges on the molecular surface (17). Among the five API types, API-160 has the highest zeta potential value, whereas API-40 has the lowest value. Therefore, the stability of different API solutions was in the order of API-160 > API-120 > API-80 > API-200 > API-40. The increase in the zeta potential could be attributed to the unfolding of protein structures, the relative migration of certain polar groups from protein interiors to surfaces, and the redistribution of surface ions (38). Therefore, it was inferred that an appropriate temperature increase helps improve the stability of the API solution.

The particle size distribution of plant protein isolates directly affects their functional characteristics and processing adaptability by regulating their solubility, interfacial activity, and structural stability (16). Figure 2B presents the particle sizes of different API solutions. The particle size distribution was concentrated between 338.52 nm (API-160) and 612.09 nm (API-200). Previous research has reported that smaller particle sizes are positively associated with higher system stability (39). Consequently, among the API solution systems evaluated in this study, the highest stability was noted for API-160, and the lowest stability was noted for API-200. This trend was consistent with the zeta potential results. Additionally, as the screw-pressing temperature increased, the main peak of the particle-sized distribution shifted to the right. A similar trend was reported for *Phaseolus vulgaris* L. protein during heat treatment (19). The possible reasons for the changes in the particle size distribution after heat treatment may be as follows. First, heat treatment can disrupt the non-covalent bonds, such as hydrogen bonds and hydrophobic interactions, that maintain the structure of protein oligomers, thereby reducing the overall particle size distribution. Second, heat treatment may increase the number of *α*-helixes and decrease the number of random coils, thereby reducing molecular flexibility. This structural ordering reduces the disordered aggregation of proteins. Third, the enhanced zeta potential and surface charge repulsion caused by heat treatment could also be the reasons (40).

#### 3.3.3 SDS-PAGE

**Figure 3:**
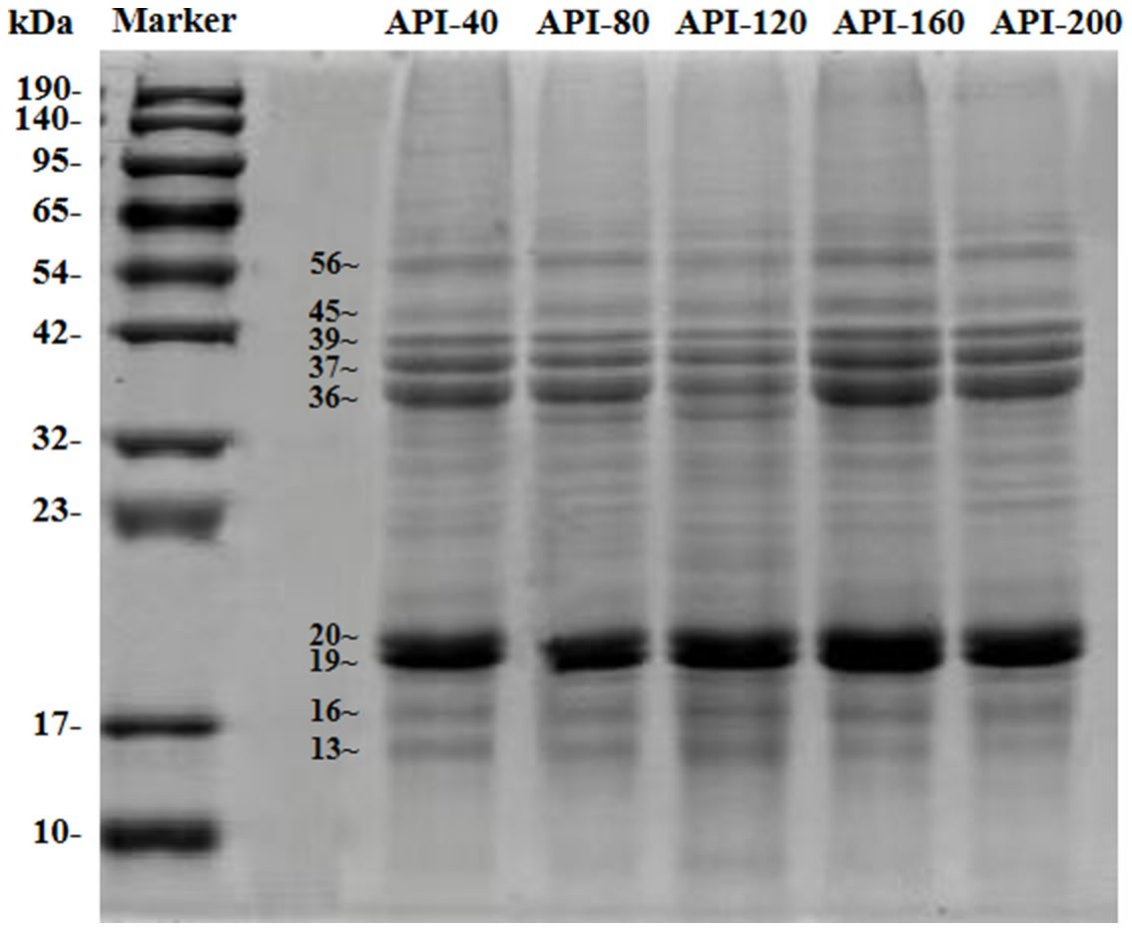
SDS-PAGE profiles of different API types.

In this study, SDS-PAGE was performed to analyze the protein profile of API, and the results are shown in Figure 3A. As shown in Figure 3, the protein bands of API were predominantly distributed in the ranges of 13–20 kDa and 36–56 kDa. In particular, the band in the 19–20 kDa region presented the highest concentration. Additionally, the subunit bands of API samples prepared at different pressing temperatures were different in color, but no obvious difference was observed. This indicated that the composition of the proteins and subunits remained almost unchanged, which means that the primary structure of the proteins did not change and that no covalent changes occurred (41). This result was consistent with that of a previous study, which reported that most bands remained unchanged when almond protein was subjected to dry heat (100°C, 200°C, and 250°C) (42).

#### 3.3.4 CD spectroscopy

**Figure 4:**
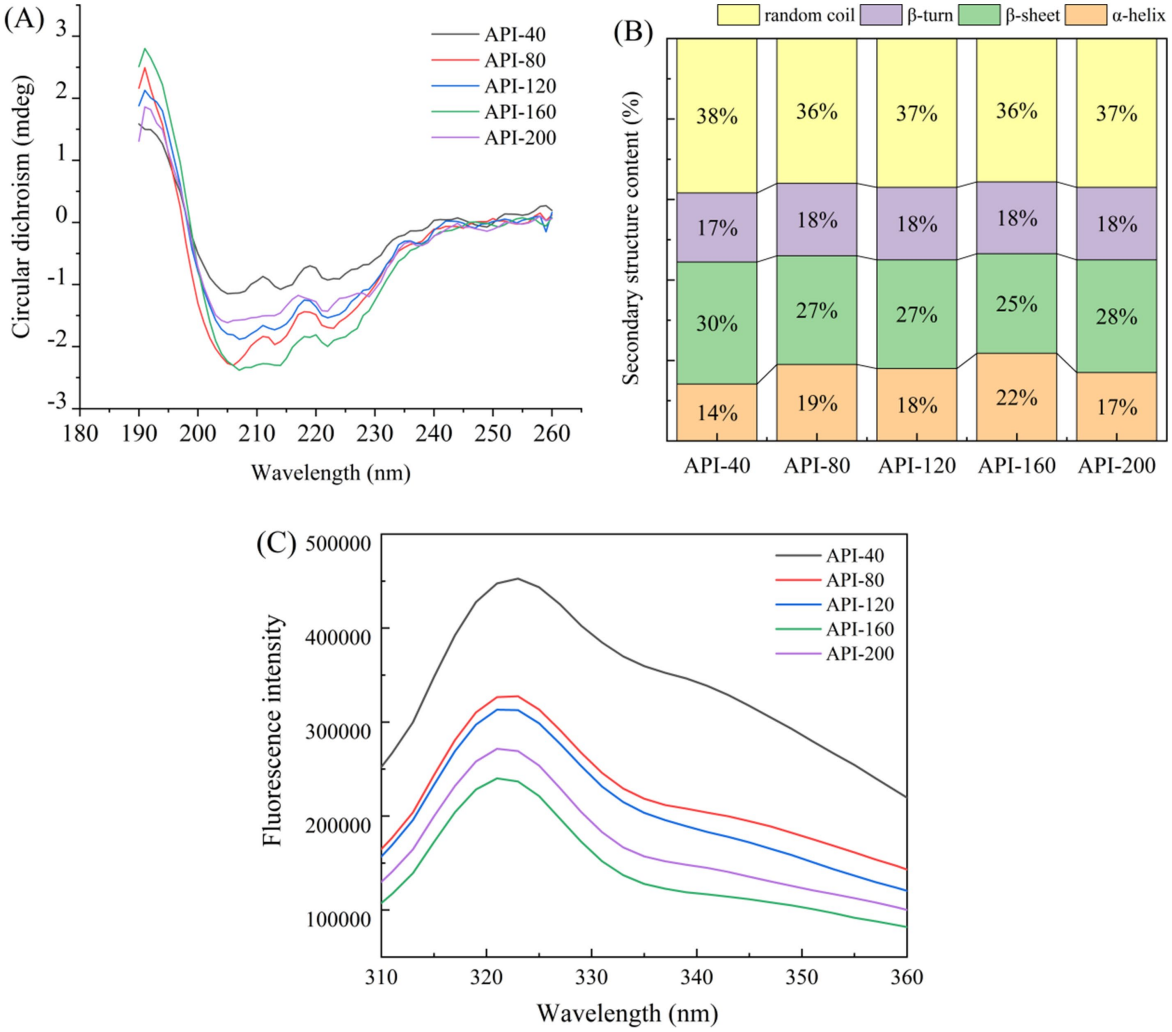
Effect of different screw-pressing temperatures on the structural characteristics of API. **(A)** CD spectroscopy, **(B)** Secondary structure content, **(C)** Fluorescence spectra.

CD spectroscopy was conducted to investigate the impact of various screw-pressing temperatures on the secondary structural properties of API. As depicted in Figure 4A, with increasing screw-pressing temperature, the number of *β*-sheets gradually decreased, whereas the ratios of *α*-helices, *β*-turns, and random coils varied for the five API types.

As shown in Figure 4A, significant changes in the peak intensities of CD spectroscopy were noted at about 205 nm and 220 nm. This could be due to plane-polarized light being absorbed differently during scanning because of the effects of insoluble particles on light scattering (16). The negative peak intensity of API-160 was significantly lower than that of API-40, whereas there was no significant change in the negative peak intensity of the API-80, API-120, and API-200 samples. The screw-pressing temperature influenced the secondary structure of a protein, and some ordered structures (such as *β*-sheets and β-turns) were retained (43). Similar findings were reported for soybean protein isolates extruded at various temperatures (43).

As shown in Figure 4B, in all five API types, random coils (36–38%) and β-sheets (25–30%) constitute a relatively high proportion of the secondary structure content, followed by *α*-helices (14–22%) and β-turns (17–18%). This result is analogous to the secondary structure of the gluten protein isolate from camellia cake reported in a previous study (15). After extrusion, the number of β-sheets in the API structure significantly decreased, whereas the number of α-helices markedly increased (*p* < 0.05). Previous studies have reported similar results in terms of how extrusion affects the physicochemical properties of lupin proteins (44). The hydrogen bonds in the α-helix are formed mainly between the amino acids inside the helix, whereas the hydrogen bonds in the β-fold are formed between amino acids in different chains. During the transformation process, these hydrogen bonds need to be rearranged (9). It is reported that heat treatment can increase the number of α-helices and decrease the number of random coils. This change can reduce the disordered aggregation and molecular flexibility of proteins, thereby increasing the stability of the protein mixture (40).

#### 3.3.5 Fluorescence spectrum analysis

The intrinsic fluorescence of the Trp, Tyr, and Phe residues in proteins, which are sensitive to different microenvironmental perturbations, serves as a probe for monitoring protein tertiary structure changes (45).

Figure 4C shows the fluorescence spectra of the API samples obtained at different screw-pressing temperatures. The maximum fluorescence intensities of the five API types were 458,300 cnt (API-40), 331,000 cnt (API-80), 316,800 cnt (API-120), 277,100 cnt (API-200), and 244,700 cnt (API-160). The fluorescence intensity of the hot-pressed API sample was lower than that of the control sample. Moreover, as the temperature increased, the fluorescence intensity of the API samples gradually decreased. The highest fluorescence peak of API was clearly redshifted, suggesting that the Trp residue’s surroundings had changed and that the structure of API had gradually unfurled (16). Similarly, the fluorescence intensity of hot-pressed cottonseed protein isolate (CPI) was lower than that of cold-pressed CPI because hot-pressed CPI has a high content of denatured protein structure (46). It was inferred that the steric conformation of the protein opened at high temperatures (such as API-160 and API-200), and the internal aromatic groups with luminescent functions were exposed, resulting in fluorescence quenching and a decreased fluorescence intensity (47). Obviously, the screw-pressing temperature induces changes in protein tertiary structure and protein aggregation. This result was also verified by the changes in the carbonyl and free sulfhydryl groups and protein surface hydrophobicity (Table 1).

#### 3.3.6 Scanning electron microscopy (SEM)

**Figure 5:**
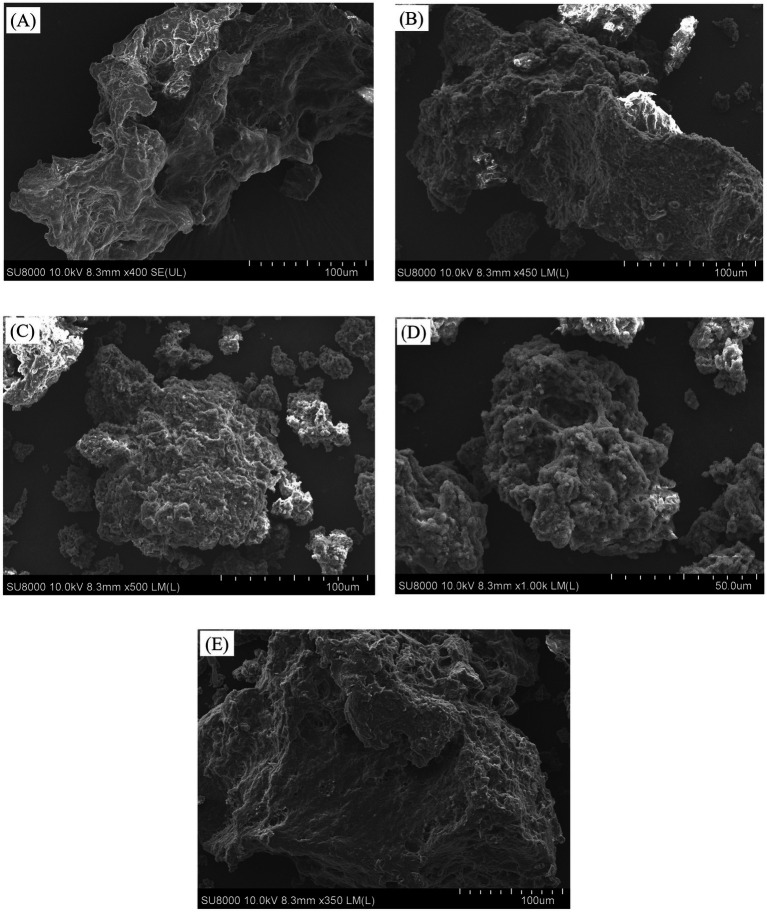
The appearance and SEM images of the API samples obtained at different screw-pressing temperatures. **(A-E)** Respectively represent API-40, API-80, API-120, API-160, and API-200.

The morphologies of the different API types are depicted in Figure 5. The degree of aggregation and dispersion conditions of the proteins may be deduced from their microstructure.

The control sample (API-40) had a relatively smooth surface and an irregular block-like structure with a few visible bubble holes. This may be due to the increase in pressure during the pressing process, which favors bubble formation (48). In contrast, the API samples obtained after hot pressing exhibited rough, porous, and irregular structures. Studies have shown that the surface structure of proteins is altered during extrusion due to heating, shearing, and pressure, resulting in a porous microstructure (15). In particular, a few small aggregates were scattered on the surface of API-80, and the surfaces of API-120 and API-160 also had irregular branching structures with small aggregates outside the branches. Similar results have been reported for corn gluten meal (49) and quinoa albumin (7), and the authors speculated that this phenomenon possibly arises from the interaction of proteins or the association of starch granules with proteins (7, 49). API-200 had a porous and multilayered structure. These findings suggested that the screw-pressing temperature changed the surface aggregation microstructure of API, which is closely related to its physicochemical and functional properties.

### 3.4 Multivariate statistical analysis

**Figure 6:**
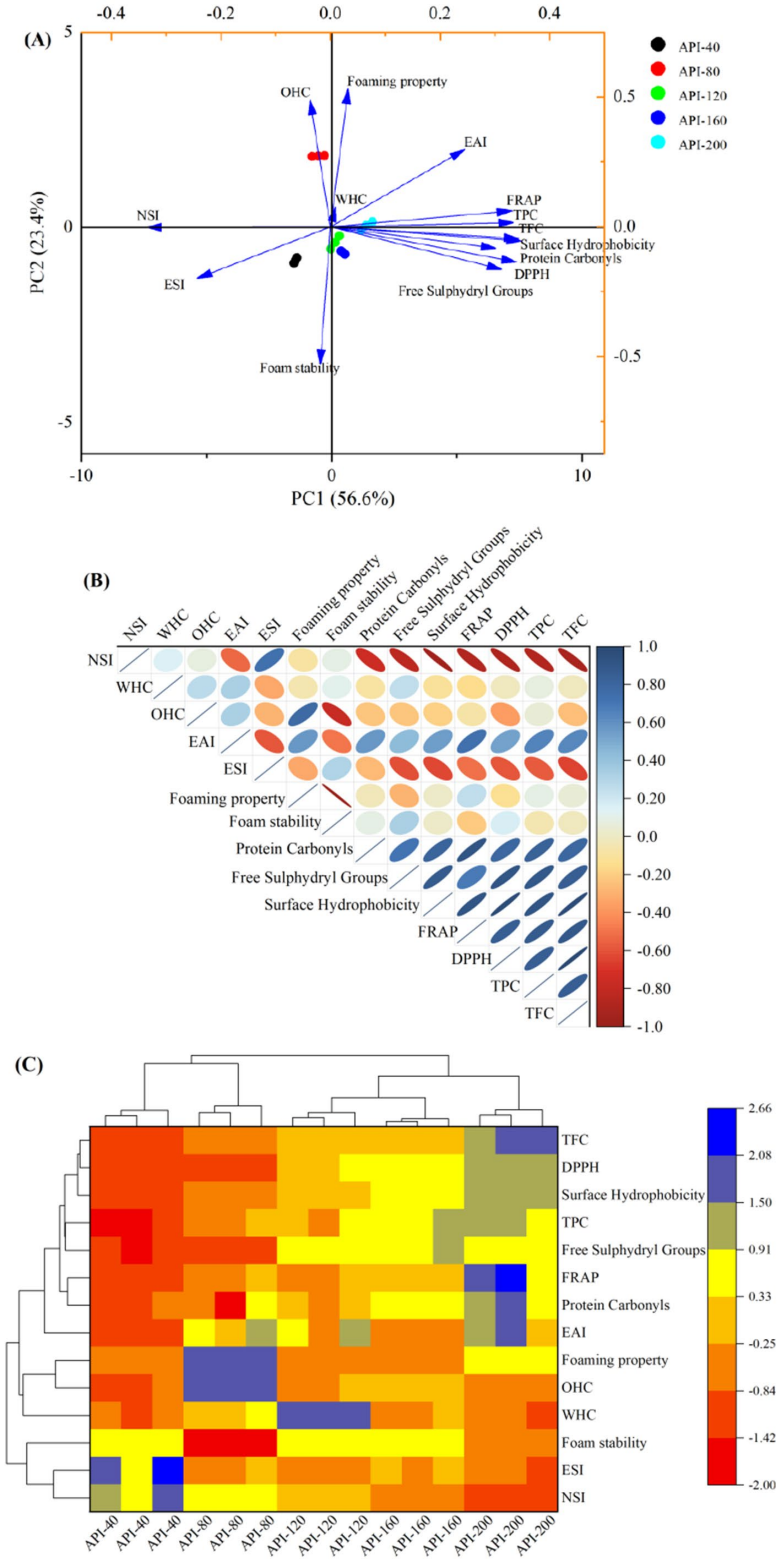
Principal component analysis **(A)**, correlation analysis **(B)**, and hierarchical cluster analysis **(C)** of different API types.

Principal component analysis (PCA) (Figure 6A), correlation analysis (Figure 6B), and hierarchical cluster analysis (Figure 6C) were conducted to investigate the relationships between the physicochemical and functional properties of different API samples.

As shown in Figure 6A, PC1, and PC2 accounted for 56.60 and 23.40% of the total variance, respectively, together explaining 80.00% of the total variation. The differences between the five API types were evident in the following two aspects. The lower right and left quadrants, i.e., the third and fourth quadrants, contained API-40, API-120, and API-160. API-80 was situated in the top left quadrant, whereas API-200 was located in the upper right quadrant. The degree of concentration and dispersion of variables on the scoring plot indicates the magnitude of differences in the physicochemical properties (16). Figure 6B shows the correlation of the detected parameters. The antioxidant activities (DPPH and FRAP) exhibited a strong positive correlation to the following parameters: protein carbonyls, surface hydrophobicity, free sulfhydryl groups, hydrophobicity values, TPC, TFC, and EAI. In contrast, NSI and ESI were negatively correlated to the above parameters. Figure 6C displays the heatmap generated in the hierarchical cluster analysis. The five API types were distributed into two clusters, separated at a distance of 17.99. API-40 and API-80 were included in Cluster 1, and the other three API types were included in Cluster 2. At a distance of 5.92, the five API types were divided into five clusters. API-40 presented higher ESI and NSI values, while most of the other tested indicators were lower than those observed for the other API samples. API-200 exhibited increased EAI, TPC, TFC, and antioxidant activity.

In summary, appropriate increases in the screw-pressing temperature helped improve the antioxidant activity, foaming capacity, and emulsification capacity of API, thereby increasing its potential for application in the food industry. On the basis of these findings, it was preliminarily concluded that pressing at 120°C was better for obtaining high-quality API. However, several limitations remain, warranting further investigation. First, expanding sample collection to diverse geographical origins and cultivars is necessary to clarify quality variations. Second, a systematic comparison of the extraction methods (including oil and protein extraction) is needed to evaluate their impacts on the physicochemical properties of API. Third, protein modification strategies (physical, chemical, or enzymatic treatments) should be explored to enhance the functional and biological characteristics of API and thereby broaden the application prospects of API in the field of healthy food.

## 4 Conclusion

This study examined the effects of the screw-pressing temperature on the functional and structural properties of API. The results showed that moderate screw-pressing temperatures (80–160°C) significantly improve the functional properties (WHC, OHC, EAI, and foaming properties) and antioxidant capacity of API. API consists of several peptides with molecular weights in the ranges of 13–20 kDa and 36–56 kDa. High-temperature pressing causes a decrease in fluorescence intensity and changes in secondary structures; specifically, the *α*-helix content increased by 4–8%, and the *β*-sheet content decreased by 2–5% in this study. The hot-pressed API has a rough, porous, and irregular structure, with small aggregates scattered on its surface. Multivariate statistical analysis clarified the relationships between the different variables of API. Accordingly, it is stated that screw-pressing temperature has a significant effect on the structural, chemical, and morphological functions of API. Therefore, during API production, temperature should be considered when high-quality protein products are to be obtained.

## Data Availability

The original contributions presented in the study are included in the article/Supplementary material, further inquiries can be directed to the corresponding author.
