# Supplementary material for: Effects of screw-pressing temperature on the functional properties and structural characteristics of apricot (Prunus armeniaca L.) kernel protein isolates
Source: Front Nutr. 2025 Jun 30;12:1619072. doi: 10.3389/fnut.2025.1619072 (PMC12256547; doi:10.3389/fnut.2025.1619072)
Supplement: Supplementary file 1 [file Data_Sheet_1.docx]

**Supplementary materials**

**Method S1. Functional properties of API**

**1.1. Water-holding capacity (WHC)**

The WHC of API were determined according to previously published method [1] with some modifications. Weigh 15 mL plastic centrifuge tube (M_1_). Accurately weigh 0.5g WPI (M_0_) and add it to the centrifuge tube. 10 mL of deionized water was added into the tube and adjust pH to 7. Vortex at 2500 rpm for 3 min to mix well. Incubate in a water bath at 50 °C for 30 min. Centrifuge at 5000 rpm for 10 min, discard the supernatant and weigh the centrifuge tube (M_2_). The formula for calculating the WHC of WPI was as follows:


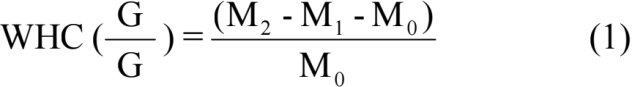


**1.2. Oil-holding capacity (OHC)**

The OHC of API was evaluated according to previously published method [1] with some modifications. Weigh a 15 mL plastic centrifuge tube (M_1_). Accurately weigh 0.5g API (M_0_) and transfer it to the centrifuge tube. 5 mL of first-grade refined soybean oil was added to the tube. Vortex at 2500 rpm for 3 min to mix well. Incubate in a water bath at 50 °C for 30 min. Centrifuge at 5000 rpm for 10 min discard the supernatant and weigh the centrifuge tube (M_2_). The formula for calculating the OHC of WPI was as follows:


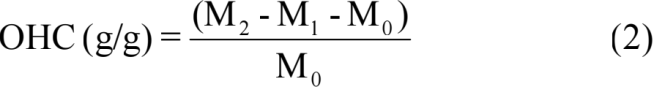


**1.3. Determination of NSI**

The content of NSI was determined using the BCA Protein Assay Kit (Enhanced Type).

The specific experimental steps are as follows: Accurately weigh 100 mg of API and dissolve it in 10 mL of 0.1 mol/L PBS solution with a pH of 7.0. Stir magnetically for 3h, then centrifuge at 8000 rpm at room temperature for 20 minutes, and take the

supernatant. According to the detection limit, the sample was diluted 28 times with 0.9% NaCl solution. The protein content of the supernatant was determined by the diquinolinic acid (BCA) method, and the absorbance of the obtained sample was substituted into the standard curve.

The standard curve of soluble protein content obtained from the reaction is:

$\text{y = 1.0077x + 0.1801 ， (R}^{2}\text{ = 0.9986)}$ (3).

**1.4. Emulsifying activity index and emulsifying stability index**

The emulsifying activity index (EAI) and emulsifying stability index (ESI) were determined according to a method described previously [2] with some modi-fications. A protein solution of 50 mL and 5 mL soybean oil were mixed together, emulsified with a XHF-DY homogenizer (Ningbo Xinzhi Biotechnology Co., LTD, China) at 15000 r/min for 2 min, 25 μL of emulsion from the bottom of the beaker was mixed with 5 mL 1 g/L SDS, and the absorbance was recorded at 500 nm for 0 (A0), 30 (A30). The EAI and ESI were calculated as follows:

[EAI ( m2 / g ) = 2× T0 × A0 × D / (1- Φ) × C×104 (4)](#bookmark1)

[ESI (%) = At /A30 ×100 (5)](#bookmark2)

where T0 is the conversion ratio (2.303), D is the dilution factor (50), C is the initial protein concentration, and Φ is the volume fraction of oil in the emulsion (0. 12).

**1.5. Foaming capacity**

FC was determined by preparing API dispersion at 5 mg/mL in 1 M phosphate buffer (pH 6). These were homogenized at 9000g for 4 min using a XHF-DY homogenizer (Ningbo Xinzhi Biotechnology Co., LTD, China) [3]. The FC was calculated as Eq. (6):


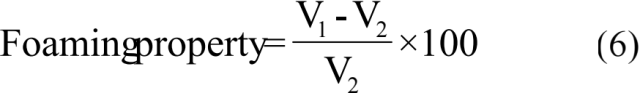


where V1 is the volume after homogenization (mL), V2 is the volume before homogenization (mL).

**Method S2. Basic structural composition 2.1. Protein carbonyls**

Protein carbonyls was determined following to previously published method [4] method with certain modifications. In this method, 4 mL of 2, 4-DNPH (20 mM in 2 M HCl) was added to 20 mg of samples, which had been appropriately dissolved into 1 mL of UP water. Subsequently, the mixture was left to stand for 1.5h at room temperature. A matched aliquot was combined with 3 mL of 2 M HCl as an absorbance blank. Five mL of 20 % (w/v) TCA was used to precipitate proteins, which were placed in an ice bath for 10 min. Blank sample was added with 4 mL of 20 % (w/v) TCA. Around 2 mL of 1:1 v/v of ethyl acetate and ethanol was used to wash the pellet three times to get rid of extra DNPH following centrifugation at 10,000g for 5 min. After that, the washed material was dissolved in 3 mL of a buffer containing 6 M GH and 1 M Na3PO4 (pH 3). Using a UV–Vis Spectrophotometer (UV2700i, Agilent Technologies CO., Ltd., America), at 375 nm protein solution’s absorbance was checked and adjusted by removing the absorbance of a blank sample. Protein carbonyl groups were determined with three repetitions for each sample. Using the Beer-Lambert equation (Eq. (7), the data were represented as nMole of carbonyl groups/mg of soluble protein, with a molar absorption coefficient for aliphatic hydrazones of 22.0 mmol/($\text{L}\bullet\text{cm}$)[5];

*A =* ε*Lc* (7)

where A is absorbance, Ԑ is molar extinction coefficient mmol/($\text{L}\bullet\text{cm}$), L is length of path (cm), and c is carbonyl-protein molar concentration (mg/mL).

**2.2. Free sulphydryl content**

Free sulfhydryl content was determined according to the method published [6] with some changes. Absorb 1 mL API solution and add it to 2.5 mL Tri-Gly buffer (0.086 mol/L Tri-0.09 mol/L Glycine-4 mmol/L Na2EDTA, pH 8.0). Then 0.1 mL of Ellman reagent (0.01 mol/L) was added. After full reaction, the supernatant was centrifuged at 6000×g for 10 min. The absorption value of the supernatant was

measured at 412 nm by ultraviolet spectrophotometer. The solution without Ellman reagent was used as the control, and the blank value was determined. Each group of samples to be tested was repeated for 3 times, and the average value was the final result.

**2.3. Surface hydrophobicity**

Surface hydrophobicity (H0) for API were determined using the hydrophobic fluorescence probe, 8-ani- lino-1-naphthalenesulfonic acid (ANS), according to the modified method of [6]. The protein –SH was determined by ANS fluorescence scanning method. The samples were prepared with Tris- HCl buffer solution of pH 8.0 into 10 mmol/L solutions of different concentrations (0.05–0.4 mg/mL). Then 4 mL of each concentration of sample solution was taken, and 20 μl of 8 × 10-3 mol/L ANS fluorescent dye stored in a light-proof environment was added, and the reaction was allowed to stand for 3 min after thorough shaking and mixing, and then measured by a fluorescence spectrophotometer (FLS920, Edinburgh Company, UK). The excitation wavelength was adjusted to 360 nm, the emission wavelength was 470 nm, the slit width was 5 nm, the protein concentration was taken as the horizontal coordinate, the fluorescence intensity was taken as the vertical coordinate, and the slope at the beginning of the curve was taken as the final H0 of the surface.

**2.4. Amino acid composition**

The amino acid composition of protein was determined with the method by Brishti et al. [7]. The protein sample was added to HCl (10 mL, 6 mol/L) with phenol (3 drops) into a reaction tube. Then, the tube was aerated with nitrogen gas for 15 min, sealed and incubated in an oven at 110℃ for 22 h. The reaction tube was cooled at room temperature and filtered through cellulose filter paper. The filtrate was dried completely using an evaporator. After that, sample dilution buffer (sodium citrate buffer, pH 2.2) was added. The solution was filtered through a 0.22 μm syringe filter prior to amino acid analysis using an automatic amino acid analyzer (LA8080, Hitachi Co. Ltd., Chiyoda-ku, Tokyo, Japan).

**References**

1. Sun, Y.; Zhong, M.; Zhao, X.; Song, H.; Wang, Q.; Qi, B.; Jiang, L. Structural and interfacial characteristics of ultrasonicated

lipophilic-protein-stabilized high internal phase Pickering emulsions. *LWT* **2022**, *158*, 113160, doi:<https://doi.org/10.1016/j.lwt.2022.113160>.

2. Yu, W.; Zhong, H.; Fang, X.; Du, M. Physicochemical properties and antioxidant potential of protein isolate from camellia cake (Camellia oleifera Abel.): Effect of different processing techniques on industrial scale. *LWT* **2023**, *184*, 114993, doi:<https://doi.org/10.1016/j.lwt.2023.114993>.

3. Rout, S.; Srivastav, P.P. Modification of soy protein isolate and pea protein isolate by high voltage dielectric barrier discharge (DBD) atmospheric

cold plasma: Comparative study on structural, rheological and techno-functional characteristics. *Food Chemistry* **2024**, *447*, 138914, doi:<https://doi.org/10.1016/j.foodchem.2024.138914>.

4. Zhang, M.; Fan, L.; Liu, Y.; Li, J. A mechanistic investigation of the

effect of dispersion phase protein type on the physicochemical stability of water–in–oil emulsions. *Food Research International* **2022**, *157*, 111293, doi:<https://doi.org/10.1016/j.foodres.2022.111293>.

5. Chen, Y.; Yao, M.; Yang, T.; Fang, Y.; Xiang, D.; Zhang, W. Changes

in structure and emulsifying properties of coconut globulin after the atmospheric pressure cold plasma treatment. *Food Hydrocolloids* **2023**, *136*, 108289, doi:<https://doi.org/10.1016/j.foodhyd.2022.108289>.

6. Wang, P.; Wang, Y.; Du, J.; Han, C.; Yu, D. Effect of cold plasma

treatment of sunflower seed protein modification on its structural and functional properties and its mechanism. *Food Hydrocolloids* **2024**, *155*, 110175, doi:<https://doi.org/10.1016/j.foodhyd.2024.110175>.

7. Brishti, F.H.; Chay, S.Y.; Muhammad, K.; Ismail-Fitry, M.R.; Zarei, M.; Saari, N. Texturized mung bean protein as a sustainable food source:

Effects of extrusion on its physical, textural and protein quality. *Innovative Food Science & Emerging Technologies* **2021**, *67*, 102591, doi:<https://doi.org/10.1016/j.ifset.2020.102591>.
